# Assessment of gray and white matter structural alterations in migraineurs without aura

**DOI:** 10.1186/s10194-017-0783-5

**Published:** 2017-07-21

**Authors:** Jilei Zhang, Yi-Lan Wu, Jingjing Su, Qian Yao, Mengxing Wang, Ge-Fei Li, Rong Zhao, Yan-Hui Shi, Ying Zhao, Qiting Zhang, Haifeng Lu, Shuai Xu, Zhaoxia Qin, Guo-Hong Cui, Jianqi Li, Jian-Ren Liu, Xiaoxia Du

**Affiliations:** 1 Shanghai Key Laboratory of Magnetic Resonance and Department of Physics, School of Physics and Materials Science, East China Normal University, 3663 North Zhong-Shan Road, 200062 Shanghai, People’s Republic of China; 2 Department of Neurology and Jiuyuan Municipal Stroke Center, Shanghai Ninth People’s Hospital, Shanghai Jiao Tong University School of Medicine, 639 Zhizaoju Road, 200011 Shanghai, People’s Republic of China; 3 Clinical Research Center, Shanghai Jiao Tong University School of Medicine, Shanghai, 200011 China

**Keywords:** Migraine without aura, Voxel-based morphometry, Surface-based morphometry, Diffusion tensor imaging, Magnetic resonance imaging

## Abstract

**Background:**

Migraine constitute a disorder characterized by recurrent headaches, and have a high prevalence, a high socio-economic burden and severe effects on quality of life. Our previous fMRI study demonstrated that some brain regions are functional alterations in migraineurs. As the function of the human brain is related to its structure, we further investigated white and gray matter structural alterations in migraineurs.

**Methods:**

In current study, we used surface-based morphometry, voxel-based morphometry and diffusion tensor imaging analyses to detect structural alterations of the white matter and gray matter in 32 migraineurs without aura compared with 32 age- and gender-matched healthy controls.

**Results:**

We found that migraineurs without aura exhibited significantly increased gray matter volume in the bilateral cerebellar culmen, increased cortical thickness in the lateral occipital-temporal cortex, decreased cortical thickness in the right insula, increased gyrification index in left postcentral gyrus, superior parietal lobule and right lateral occipital cortex, and decreased gyrification index in the left rostral middle frontal gyrus compared with controls. No significant change in white matter microstructure was found in DTI analyses.

**Conclusion:**

The significantly altered gray matter brain regions were known to be associated with sensory discrimination of pain, multi-sensory integration and nociceptive information processing and were consistent with our previous fMRI study, and may be involved in the pathological mechanism of migraine without aura.

## Background

Migraine constitute a disorder characterized by recurrent headaches of moderate to severe intensity, pulsating quality, and unilateral location, that are aggravated by routine physical activity and associated with nausea, photophobia, and/or phonophobia [1]. It has been demonstrated that migraine have a high prevalence and a high social-economic burden, and they severely affect quality of life. In recent years, neuroimaging technology has provided more convenient methods for better understanding the pathological mechanism of migraine and identifying abnormal brain regions associated with migraine.

Several MRI studies have identified functional and structural changes between migraine patients and healthy controls, and have suggested that brain malfunctioning may be associated with migraine pathophysiology [2–7]. In addition, repeated and long-term migraine attacks may induce functional and structural plastic changes that may underlie the progression of the disorder [8, 9]. Voxel-based morphometry (VBM) and surface-based morphometry (SBM) are advantageous for evaluating structural alterations (such as gray matter volume, cortical thickness and gyrification index [GI]) of the gray matter due to their ability to localize abnormal brain regions in patients without a priori hypothesis [10–12]. Two previous meta-analyses aimed to locate concordant gray matter alterations in migraine patients and found concordant decreases in gray matter volume (GMV) in some brain regions involved in pain-related processes [13, 14]. The patient groups of previous VBM studies have been primarily migraineurs without aura, but other subtypes (such as migraineurs with aura, and patients with chronic migraine) were also included. Previous research has proposed that the different subtypes of migraine may present specific structural alterations [15, 16]. In addition, diffusion tensor imaging (DTI) has been extensively used to evaluate the microstructural changes in white matter based on the diffusion characteristics of water molecules in the brain [17]. Migraineurs exhibited several microstructural alterations in previous DTI studies [18–21]. Conversely, Need et al. did not identify microstructural white matter alterations in chronic and episodic migraine patients [22]. The results of structural studies of migraine patients seem contradictory and inconsistent, and the patients groups are characterized sample heterogeneity. Thus, structural alterations of the white matter and gray matter in migraineurs without aura should be further investigated.

Our previous studies detected dysfunction in various brain regions in migraineurs based on task functional magnetic resonance imaging (fMRI) and task-free fMRI [8, 23, 24]. We found activation in the visual cortex and anterior cerebellum lobe/culmen during presentation of negative emotion picture stimuli in migraineurs [23]. Furthermore, migraineurs without aura exhibited dysfunction in the default mode network and sensorimotor network during the task-free state [8, 24].It has been demonstrated that the function of the human brain is intimately related to its structure [25, 26]. Functional abnormalities in migraineurs may be caused by corresponding structural changes. Therefore, we used SBM, VBM and DTI analyses to detect structural alterations of the white matter and gray matter in 32 migraineurs without aura in current study. In addition, we hypothesized that migraine patients without aura may exhibit structural changes and that these changes may be consistent with the findings of previous fMRI studies and associated with the pathological mechanisms of migraine.

## Methods

The East China Normal University Committee on Human Research (Project No. HR2016/03022) and the Independent Ethics Committee of Shanghai Ninth People’s Hospital (Project No. [2016]01), Shanghai Jiao Tong University School of Medicine, approved the current study. All migraineurs without aura and healthy controls provided written informed consent using forms approved by the committee.

### Subjects

Thirty-two migraineurs without aura (8 males, 24 females) were recruited from among the outpatients of the Department of Neurology at Shanghai Ninth People’s Hospital. These patients were diagnosed with migraine by a neurologist based on the International Classification of Headache Disorders (ICHD-III beta, 2013) [1]. During the interview, the neurologist also obtained the migraineurs’ demographic and clinical data, including age, sex, disease duration, attack frequency (times/month), and attack duration (hours), and their scores on the visual analogue scale (VAS), the Migraine Disability Assessment Scale (MIDAS) and the Headache Impact Test (HIT-6). Migraineurs reported that they had no headaches 48 h before MRI scans and did not suffer a migraine attack or discomfort during the MRI scans. In the current study, patients were excluded if they suffered chronic migraine or were taking preventive medication. Thirty-two age- and gender-matched healthy controls (8 males, 24 females) who had not experienced any headaches or chronic pain disorders in the past year and whose family members did not suffer from migraine or other headaches were recruited. All subjects reported right-handed and had no substance abuse, and all neurological and psychiatric diseases were excluded based on clinical examination and a structured interviews. The details are provided in Table 1.

**Table 1:** Demography and clinical scores of the migraine group and control group

	Migraine group (Mean ± SD)	Control group (Mean ± SD)
Male/Female	8/24	8/24
Age(years)	38.3 ± 10.16	38.8 ± 10.02
Disease duration(years)	9.5 ± 6.23	-
Attack duration (hours)	20.5 ± 20.02	-
Attack frequency (times/months)	3.36 ± 2.55	-
VAS	7.3 ± 2.04	-
MIDAS	13.2 ± 20.53	-
HIT-6	62.8 ± 10.04	-

*Abbreviations*: *VAS* visual analogue scale, *MIDAS* Migraine Disability Assessment Scale, *HIT-6* Headache Impact Test, − no data

### MRI acquisition

DTI and high-resolution T_1_-weighted MRI data were acquired using a 3.0 Tesla Siemens Trio Tim MRI scanner with a 12-channel head coil at the Shanghai Key Laboratory of Magnetic Resonance (East China Normal University, Shanghai, China). Custom-fit foam pads were used to minimize head movement of the subjects. The parameters of the pulse sequence were as follows: 1) high-resolution T1-weighted 3-dimensional magnetization-prepared rapid-acquisition gradient-echo pulse sequence, repetition time = 2530 ms, echo time = 2.34 ms, inversion time = 1100 ms, flip angle = 7°, number of slices = 192, sagittal orientation, field of view = 256 × 256 mm^2^, matrix size = 256 × 256, and slice thickness = 1 mm. 2) The DTI acquisition utilized a single-shot spin-echo echo planar imaging sequence in the contiguous axial plane, repetition time = 8900 ms, echo time = 86 ms, b-value = 0 and 1000 s/mm2, slice thickness = 2 mm, and 70 slices, matrix size = 128 × 128, field of view = 256 × 256 mm^2^, diff direction = 64, and the resolution = 2 × 2 × 2 mm^3^.

### VBM and SBM analysis

The VBM and SBM analysis were conducted using the Computational Anatomy Toolbox (CAT12, http://dbm.neuro.uni-jena.de/cat/) that is an extension toolbox of Statistical Parametric Mapping software (SPM12, http://www.fil.ion.ucl.ac.uk/spm/software/spm12). We used the default settings that are described in detail in the manual of the CAT 12 toolbox (http://dbm.neuro.uni-jena.de/cat12/CAT12-Manual.pdf). The T1 images were spatially registered to the Montreal Neurological Institute (MNI) template. Then, the whole brain structural data were segmented into white matter, gray matter and cerebrospinal fluid. Bias correction was performed to remove intensity non-uniformities. Segmented images of the gray matter were preserved to assess the amount of volume changes based on spatial registration, and the modulated images of the gray matter could reflect the tissue volumes for using VBM analysis. The total intracranial volume (TIV) of each subject was calculated and used as a covariate for further statistical analyses. Finally, the normalized gray matter images were smoothed using a Gaussian filter (8 mm full-width half-maximum, FWHM).

CAT 12 provides a fully automated method to estimate cortical thickness and the central surface of hemispheres based on the projection-based thickness method [11]. The GI were extracted from central surface data based on the absolute mean curvature as previously described [12]. The cortical thickness and GI images of the left and right hemispheres were smoothed with a 15-mm FWHM Gaussian kernel.

### DTI analyses

The analysis of DTI images was conducted using FSL v5.0 (http://fsl.fmrib.ox.ac.uk/fsl/fslwiki) and SPM 12 software. First, “eddy current correction” was implemented to correct for head motion artifacts and eddy current distortions. The brain was extracted using the Brian Extraction Tool (BET v2.1) in FSL. Then, FA and first, second and third eigenvalue (L1, L2, L3) maps were calculated in the individual space by fitting a diffusion model at each voxel using DTIFIT. The AD equals L1, and the RD equals the mean values of L2 and L3. Then, normalization and statistical analyses of the FA, RD and AD maps were performed using SPM 12. We co-registered the high-resolution T_1_-weighted image to B_0_ images. The T_1_ images were then segmented into gray matter and white matter and generated bias-field corrected structural image and deformation fields. The FA, RD and AD maps were spatially normalized to the standard Montreal Neurological Institute (MNI) stereotaxic space and resampled to 2 × 2 × 2 mm^3^. Finally, spatial smoothing was performed with an 8 mm FWHM Gaussian kernel.

### Statistical analysis

The GMV was assessed by voxel-wise two-sample t-tests within the brain mask with TIV as a covariate to correct for different brain sizes among subjects. The cortical thickness and GI maps of the left and right hemispheres, and the FA, MD, AD and RD maps were separately statistically analyzed within a brain mask using voxel-wise two-sample t-tests. To address multiple comparisons, all statistical maps were assigned thresholds at *p* < 0.005 (voxel level), and the false discovery rate (FDR) was corrected to *p* < 0.05 at the cluster level. The surviving clusters were reported.

## Results

### Demographics

The demographic and clinical data of the migraine and control groups are presented in Table 1. The age and gender demographic factors did not significantly differ between the migraineurs without aura and controls.

### VBM and SBM

**Fig. 1:**
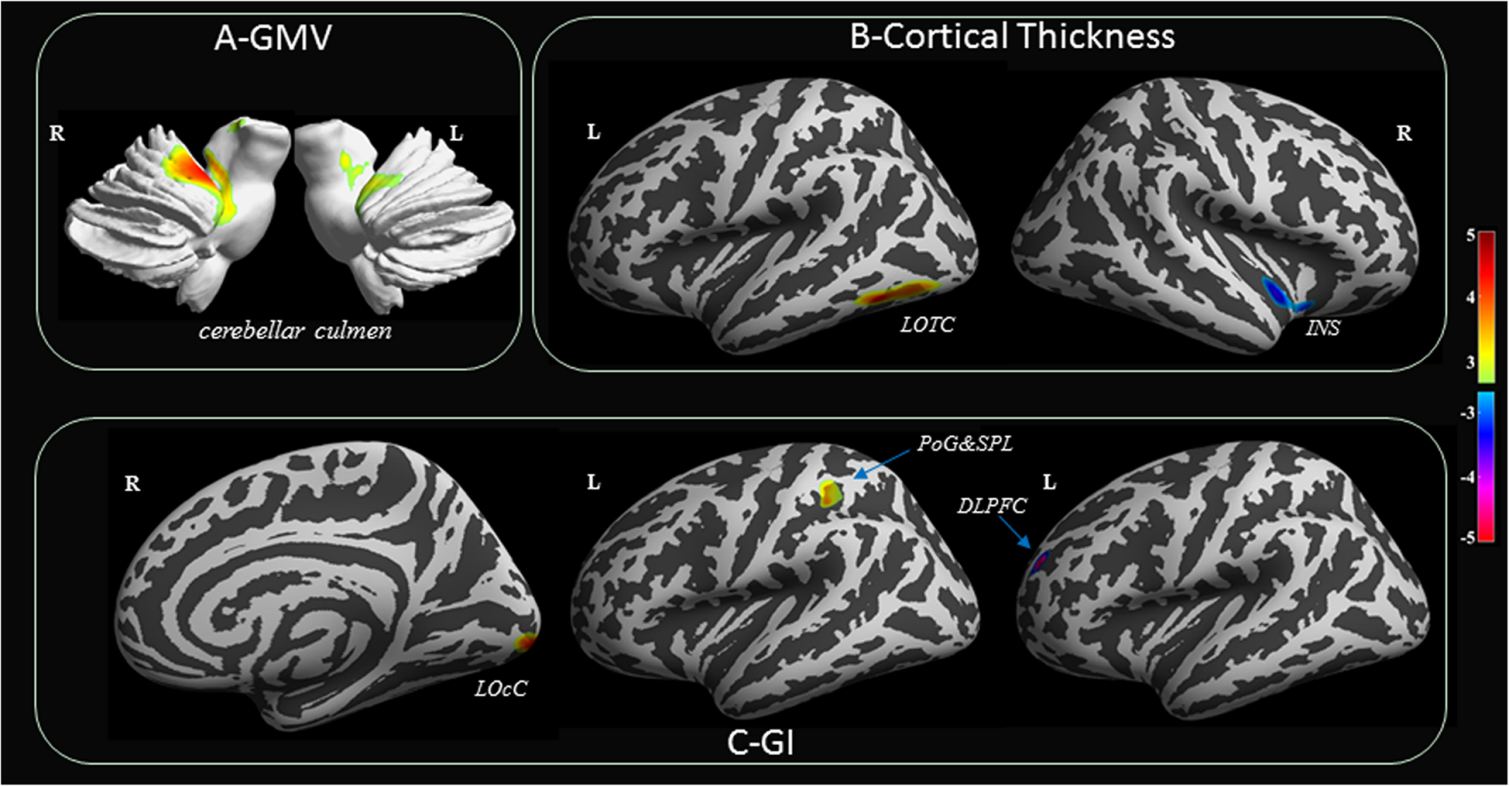
The structural differences in gray matter between migraineurs without aura and healthy controls. a, compared with controls, the migraineurs without aura exhibited significantly increased gray matter volume in bilateral cerebellar culmen (lobule I-IV and lobule V). b, cortical thickness was significantly thicker in the left LOTC, and significantly thinner in the right INS in migraineurs without aura than in the healthy controls. c, the GI were significantly increased in the left POG, the SPL and the right LOcC, and decreased in the left DLPFC in the migraineurs. *LOTC* lateral occipital-temporal cortex, *INS* insula, *POG* postcentral gyrus, *SPL* superior parietal lobule, *LOcC* lateral occipital gyrus, *GI* gyrification index, *GMV* gray matter volume

Compared with controls, the migraineurs without aura exhibited significantly increased gray matter volume in bilateral cerebellar culmen (lobule I-IV and lobule V) extending to the lingual gyrus, thalamus, fusiform and parahippocampa gyrus (Fig. 1a). Cortical thickness was significantly thicker in the left inferior temporal and lateral occipital cortex, and significantly thinner in the right insula in migraineurs without aura than in the healthy controls (Fig. 1b). The GI were significantly increased in the left postcentral gyrus, the superior parietal lobule and the right lateral occipital cortex, and decreased in the left rostral middle frontal gyrus in the migraineurs (Fig. 1c).

### DTI

There were not significant differences in FA, RD and AD between migraineurs without aura and healthy controls.

## Discussion

To the best of our knowledge, this is the first study to combine VBM, SBM and DTI analyses for evaluating structural and microstructural alterations of the gray matter and whiter matter in migraineurs without aura. We found that the significantly altered brain regions in migraine patients were primarily concentrated in the gray matter based on VBM and SBM analyses (Table 2, Fig. 1), whereas there were no significant microstructural changes in the white matter in DTI analyses.

**Table 2:** Significant inter-group differences in gray matter volume, cortical thickness and gyrification index between migraine patients without aura and healthy controls

Predominant regions in cluster	Cluster size	Peak T value	MNI coordinates		
			x	y	z
GMV increase in migraine group without aura					
Right cerebellar culmen extending to the right lingual gyrus, right thalamus and right parahippocampa gyrus	3742	5.77*	15	−38	−14
Left cerebellar culmen extending to left fusiform gyrus and left parahippocampal gyrus	1543	3.82	−21	−32	−15
Thickness increase in migraine group without aura					
Left inferior temporal gyrus and lateral occipital cortex	980	4.37	−48	−57	−10
		3.9	−41	−67	−7
Thickness decrease in migraine group without aura					
Right insula	653	-3.55	33	15	−13
GI increase in migraine group without aura					
Left postcentral gyrus and superior parietal lobule	601	3.67	−34	−35	40
Right lateral occipital cortex(pole occipital)	555	4.56	17	−99	−13
GI decrease in migraine group without aura					
left rostral middle frontal gyrus	308	-5.05*	−23	45	21

The results were thresholded at *p* < 0.005 (voxel level) and FDR corrected to *p* < 0.05 at the cluster level

‘*’ indicates that the peak T value passes the voxel-wise level FDR correction (*p* < 0.05). GI, gyrification index; GMV, gray matter volume

Compared with controls, migraineurs without aura exhibited significantly increased gray matter volume in the bilateral cerebellar culmen (lobule I-IV and lobule V) extending to the lingual gyrus, thalamus, fusiform and parahippocampal gyrus. The cerebellum has been recently proposed to be associated with cognitive, sensorimotor, pain and affective information processing [27–30], and to be involved in pathophysiological mechanism of migraine [31]. Moulton et al. found that the cerebellar activation areas overlapped with both unpleasant picture viewing and heat pain in healthy subjects and suggested that the cerebellum may have specific areas associated with encoding of generalized aversive processing [32]. In our previous task-fMRI study, we found that migraineurs exhibited hyperactivation in the anterior cerebellum lobe/culmen and visual cortex while viewing negative minus neutral affective pictures compared with healthy controls, and we proposed that migraine patients may have hypersensitivities to negative affective stimuli or that there is less inhibition in the cerebellum of migraineurs [23]. In addition, Mehnert J et al. demonstrated that the cerebellum, including lobules V and I-IV, is active during trigeminal nociceptive stimulation, and that the activity of the cerebellum is modulated by the perceived intensity of pain [33]. Nociceptive and negative emotion picture stimuli are parts of aversive stimuli and can active cerebellar responses. Thus, we propose that the increased GMV in the anterior cerebellum lobe (lobules V and I-IV) is consistent with our previous task-fMRI study and may be involved in the pathology mechanism of migraine.

Migraine patients exhibited increased cortical thickness in left inferior temporal gyrus and lateral occipital cortex and increased GI in the right lateral occipital cortex. It has been demonstrated that the lateral occipital-temporal cortex plays an important role in the multi-sensory integration of visual, auditory and tactile information [34, 35]. In line with our results, Messina et al. found that patients with migraine had an increased thickness of the left temporo-occipital incisure compared with control subjects [36]. The structural abnormalities of temporo-occipital cortex might be to explain the interictal deficits in visual motion processing described in migraineurs [37–39].

In our study, we found that migraineurs without aura exhibited decreased cortical thickness in the right insula, decreased GI in the left rostral middle frontal gyrus, and increased GI in the left postcentral gyrus and superior parietal lobule. In fact, it has been proposed that the postcentral gyrus, superior parietal lobule, insula and dorsolateral prefrontal cortex are involved in sensory discrimination of pain information [40–42]. Furthermore, Mehnert et al. observed increases in cerebellar-cortical connectivity in some brain regions, including insula and lingual gyrus [33], during trigeminal nociception stimuli. The postcentral gyrus and insula play important roles in the ascending trigemino-thalamo-cortical nociceptive pathway and have been implicated in the pathophysiology mechanism of migraine [3, 43]. Our previous study revealed that the bilateral postcentral gyrus is functional alterations in migraineurs without aura and that the postcentral gyrus has decreased functional connectivity with the contralateral insula, superior parietal lobule, prefrontal cortex and occipital cortex [8]. These findings suggest that structural alterations in the insula, postcentral gyrus, superior parietal lobule and rostral prefrontal cortex may disrupt the pathway used to discriminate sensory features of pain or the trigemino-thalamo-cortical pathway, and induce hypersensitivities to painful stimuli in migraineurs.

DTI analysis did not reveal any significant differences in migraineurs without aura comparing with healthy controls. Previous DTI studies in migraine patients detected several alterations, although the results of these studies are contradictory and inconsistent [18–21]. Liu et al. did not find that the migraineurs without aura exhibited significant microstructural alterations of white matter at a 1-year follow-up evaluation [44]. In line with our results, Need et al. demonstrated no microstructural white matter changes in episodic and chronic migraine patients based on Tract-based spatial statistics (TBSS) analysis [22].

## Conclusions

In the current study, we evaluated the structural alterations of white matter and gray matter in migraine patients without aura using VBM, SBM and DTI analyses. Gray matter structural alterations in migraine patients were detected in the bilateral cerebellar culmen, lateral occipital-temporal cortex, right insula, left prefrontal cortex, left postcentral gyrus and superior parietal lobule. No significant changes in white matter regions were found in the DTI analyses. Our findings are consistent with previous fMRI studies, and we propose that the significant alterations in the gray matter, which are associated with sensory discrimination of pain, multi-sensory integration and nociceptive information processing, and may be involved in the pathological mechanism of migraine without aura.

## Abbreviations

AD: Axial diffusivity
DTI: Diffusion tensor imaging
FA: Fractional anisotropy
fMRI: Functional magnetic resonance imaging
FWHM: Full-width half-maximum
GI: Gyrification index
GMV: Gray matter volume
HIT-6: Headache Impact Test
MIDAS: Migraine Disability Assessment Scale
RD: Radial diffusivity
SBM: Surface based morphometry
TIV: Total intracranial volume
VAS: Visual analogue scale
VBM: Voxel based morphometry
